# The extract of concentrated growth factor enhances osteogenic activity of osteoblast through PI3K/AKT pathway and promotes bone regeneration in vivo

**DOI:** 10.1186/s40729-021-00357-4

**Published:** 2021-08-04

**Authors:** Kai Dong, Wen-Juan Zhou, Zhong-Hao Liu, Peng-Jie Hao

**Affiliations:** Department of Dental Implantology, Yantai Stomatological Hospital Affiliated to Binzhou Medical College, No. 142, North Great Str, Yantai, Shandong 264008 People’s Republic of China

**Keywords:** Platelet concentrates, CGF, Osteoblast, Osteogenic, PI3K/AKT pathway

## Abstract

**Background:**

Concentrated growth factor (CGF) is a third-generation platelet concentrate product; the major source of growth factors in CGF is its extract; however, there are few studies on the overall effects of the extract of CGF (CGF-e). The aim of this study was to investigate the effect and mechanism of CGF-e on MC3T3-E1 cells in vitro and to explore the effect of combination of CGF-e and bone collagen (Bio-Oss Collagen, Geistlich, Switzerland) for bone formation in cranial defect model of rats in vivo.

**Methods:**

The cell proliferation, ALP activity, mineral deposition, osteogenic-related gene, and protein expression were evaluated in vitro; the newly formed bone was evaluated by histological and immunohistochemical analysis through critical-sized cranial defect rat model in vivo.

**Results:**

The cell proliferation, ALP activity, mineral deposition, osteogenic-related gene, and protein expression of CGF-e group were significantly increased compared with the control group. In addition, there was significantly more newly formed bone in the CGF-e + bone collagen group, compared to the blank control group and bone collagen only group.

**Conclusions:**

CGF-e activated the PI3K/AKT signaling pathway to enhance osteogenic differentiation and mineralization of MC3T3-E1 cells and promoted the bone formation of rat cranial defect model.

## Background

A series of cascade reactions directed to heal the damaged bone tissue, similar to fracture healing, are promptly activated once the oral implant has been inserted into the bone. It is known that platelets play a pivotal role during the above process by initiating coagulation and locally releasing growth factors [1]. In this context, the use of platelet-derived concentrates to promote bone reconstruction is of interest. Previous studies have validated that concentrated growth factor (CGF), a third-generation platelet concentrate derived from autologous blood, is essential to promote the proliferation and osteogenic differentiation of mesenchymal stem cells in vitro but also enable excellent healing of bone defects of critical sizes in vivo [2–5]. However, there is relatively little research regarding the mechanism of action of CGF.

CGF gel is a three-dimensional net-like structure interwoven with a large number of fibers and a variety of growth factors such as platelet-derived growth factor (PDGF), transforming growth factor-β (TGF-β), epidermal growth factor (EGF), bone morphogenetic protein-2 (BMP-2), vascular endothelial growth factor (VEGF), and insulin-like growth factor (IGF); furthermore, any exogenous products were not included [6]. The use of CGF has a wide range of clinical applications in the regeneration of alveolar and sinus bone; however, there is some controversy about the potential therapeutic effect of CGF [7–9]. This might be explained by the following two reasons. First, CGF gel is degraded too rapidly to maintain the long-term stable release of growth factors. Furthermore, the most major source of growth factors in CGF is its extract (CGF-e); it seems that the effective concentration of growth factors appears to be more important than fibrin structure [10]. Second, CGF contains not only growth factors favorable for bone formation but also leucocytes and inflammatory cytokines which may inhibit bone formation [11]. Thus, it is important to examine the pure effect of growth factors contained in CGF under a distraction-free setting. In addition, the potential mechanisms of CGF-mediated bone regeneration requires further clarification.

One of the pivotal signaling pathways regulating the bone regenerative process in many systems is the phosphoinositide 3-kinase (PI3K)/serine-threonine kinase (Akt) pathway which was shown to be involved in proliferation and differentiation of osteoblasts [12]. PI3K, a heterodimeric enzyme, is a critical factor for bone resorption and bone formation; once PI3K is phosphorylated, it activates Akt, which is a key downstream molecule. At present, whether the PI3K/Akt signaling pathway is involved in the regulation of the proliferation and differentiation of osteoblasts treated with CGF-e is still less investigated.

In the present study, we prepared CGF-e without any leucocytes and detected the concentration of various growth factors contained in CGF-e. And then, we explore the effect and mechanism of CGF-e on osteoblasts in vitro and to investigate the effect of combination of CGF-e and bone collagen (Bio-Oss Collagen, Geistlich, Switzerland) for bone formation in cranial defect model of rats in vivo. Osteoblast-like cell (MC3T3-E1) was a mature and stable osteogenic cell line, origins from mice parietal, which can effectively simulate the osteogenesis process. In order to evaluate whether CGF-e would induce a xeno-antigen immune rejection, we choose the MC3T3-E1 osteoblast-like cells line.

Taken together, we hypothesized that CGF-e could promote osteogenic differentiation and mineralization of MC3T3-E1 cells through the PI3K/AKT signaling pathway and could enhance the bone formation of rat cranial defect model.

## Methods

### Preparation of CGF-e

Thirty male Sprague–Dawley (SD) rats (250–300g) were used to prepare CGF. All the animal procedures were implemented in accordance with the animal ethics guidelines and were granted by the ethics committee of Yantai Stomatological Hospital (NO. 2020-003). CGF-e was prepared according to the following steps: 10ml venous blood was collected from the inferior vena cava of each SD rat and immediately centrifuged in the special equipment (Medifuge centrifuge, Italy). After centrifugation, 3 layers were observed in the blood: the upper platelet poor plasma layer, the middle fibrin-rich gel with aggregated platelets and concentrated growth factors, and the lower red blood cell layer [13]. Then, CGF gel was cut into small pieces and put in a −80°C freezer for 1h. After three cycles of freeze (−80°C) and thaw (4°C), the exudates were harvested and centrifuged at 4°C for 30 min at 230g and defined as “CGF-e”. The subsequent experiments were performed with 0.5% CGF-e [14].

### Enzyme-linked immunosorbent assay (ELISA)

Concentrations of BMP-2, EGF, PDGF-BB, TGF-β1, TNF-α, and IL-1 in CGF-e and serum were detected by ELISA, respectively. The operations were conducted strictly in accordance with the manufacturer’s instructions of commercially available ELISA kit (Solarbio, China).

### Cell culture

MC3T3-E1 cells (Shanghai Cell Bank of the Chinese Academy of Sciences) were cultured in α-MEM osteogenic induction medium (10% fetal bovine serum, 50μM ascorbic acid-2-phosphate, and 5mM β-glycerophosphate) and cultivated in an incubator that maintained the temperature at 37°C and carbon dioxide (CO_2_) at 5%. The cells were passaged by digestion with 0.25% trypsin once they reached 80–90% confluence. All experiments were repeated three times, with three replicates.

### Cell proliferation assay

Cells were seeded in 96-well plates at 1 × 10^4^ cells/well and cultured for 3 days and 7 days. For the MTT assay, 20 μL of MTT solution (5mg/mL) was added per 100 μL of media and incubated at 37°C for 4 h, followed by the addition of 100μl DMSO to dissolve MTT crystals. Absorbance at 570 nm was measured using a microplate reader (Bio-Rad, USA).

### Alkaline phosphatase (ALP) staining and activity assay

Cells were seeded in 24-well plates at 1 × 10^4^ cells/well and cultured for 3 days and 7 days. For ALP staining, cells were washed with PBS twice and then fixed with 4% paraformaldehyde for 10min; alkaline phosphatase (ALP) staining was performed using ALP staining solution (Azo coupling method, Beijing Solebao Bio, China) for 15 min at room temperature and protected from light. For activity assay, the culture supernatants were determined by measuring hydrolysis of p-nitrophenyl phosphate according to the manufacturer’s instructions (Beyotime Institute of Biotechnology, China).

### Mineralization assay

Cells were seeded in 24-well plates at 5 × 10^3^ cells/well and cultured for 21 days. Alizarin red staining was used to stain the calcium nodules. Briefly, cells were fixed with 4% paraformaldehyde for 30 min and stained with 2% Alizarin Red S (Sigma, USA) for 20 min at room temperature. For quantification, the stained calcium nodules were treated with 10% cetylpyridinium chloride solution (Sigma, USA). The absorbance was measured using a microplate reader at 570 nm (Bio-Rad, USA).

### Quantitative real-time PCR analysis

Cells were seeded in 24-well plates at 1 × 10^4^ cells/well and cultured for 7 days. Total RNA was isolated from cells by use of the RNA ex Pro Reagent Kit (AG, China), in accordance with the manufacturer’s instructions. cDNA was synthesized by use of the Evo M-MLV Kit (AG, China). The cDNA was then analyzed by real-time PCR analysis by use of the SYBR green kit (Takara, Japan) and Rotor-Gene 3000 RT PCR system (Corbett, Australia). Expression values were normalized to GAPDH. The primer sequences of BMP-2, osteocalcin (OCN), and PI3K are shown in Table 1.

**Table 1:** Polymerase chain reaction primer sequences

Target	Primer sequences (5′-3′)	Product size (bp)
*GAPDH*	F TTGTCTCCTGCGACTTCAACA	182
	R GTGGTCCAGGGTTTCTTACTCC	
*BMP-2*	F TAAGGATTAGCAGGTCTTTG	440
	R CACAACCATGTCCTGATAAT	
*OCN*	F AGGAGGGCAATAAGGTAGTGAA	165
	R TACCATAGATGCGTTTGTAGGC	
*PI3K*	F CGGTTTCTCCCTTCTACTTCCTG	83
	R GCTCTGCCTCAGCCTTTTATTG	

*GAPDH* Glyceraldehyde-3-phosphate dehydrogenase, *BMP-2* Bone morphogenetic protein-2, *OCN* Osteocalcin, *PI3K* Phosphoinositide 3-kinase

### Western blot analysis

Cells were seeded in 6-well plates at 1 × 10^4^ cells/well and cultured for 10 days. Total protein was extracted using RIPA lysis solution (Beyotime, China). Protein concentration was determined by BCA assay (Beyotime, China). Equal amounts of protein were separated using 10% SDS-PAGE and electrophoretic transfer to polyvinylidene fluoride (PVDF) membranes (Millipore, USA). After blocking with 10% milk, the blots were incubated with primary antibodies (PI3K p85 Tyr458, 1:1000, CST; AKT, 1:1000; Cell Signaling Technologies, USA; p-AKT Ser473, 1:1000; CST; GAPDH, 1:10000, Golden Bridge International, China) at 4°C overnight. Then, the cells were rinsed with PBS and incubated with secondary antibody (Golden Bridge International, China) for 3h.

### Signaling pathway inhibition

LY294002, a PI3K inhibitor, was purchased from Sigma (St. Louis, MO, USA) and dissolved in dimethyl sulfoxide (DMSO) at a stock concentration of 10mM. MC3T3-E1 cells were pretreated with the recommended concentrations of LY294002 (10μM) for 1h and subsequently supplemented with 0.5% CGF-e for co-culture; the culture medium was changed every 2 days.

### Animal experiment

Thirty Sprague-Dawley rats (8 weeks, 250–300g) were randomly assigned to three groups with 10 rats in each group: unfilled defect for control (group C), bone collagen alone (group B), and CGF-e + bone collagen (group BC). Rats in the group BC were given local subcutaneous injection of 0.5% CGF-e (0.1mL/2 days). Breeding environment of rats was set: 12 h day and night alternation, clean grade, room temperature 20–25°C, humidity 55–60%, free food and drinking water, as well as adaptable feeding for 1 week. All surgical procedures involving animals were implemented according to the animal ethics guidelines and were granted by the ethics committee of Yantai Stomatological Hospital (NO. 2020-004). According to Spicer et al. [15], two circular 5-mm diameter calvarial defects were made with a trephine bur. The skin and periosteum were respectively closed using absorbable sutures (5-0, Vicryl®, Ethicon, USA). Rats were sacrificed, and the calvaria samples were harvested on postoperative days 14 and 42, respectively, and fixed in 4% formaldehyde at 4°C.

### Histologic assessment

The calvaria samples were decalcified in 10% EDTA at pH 7.4 for 2 months. After decalcification, the sample was embedded in paraffin and cut into 5-μm thick serial sagittal sections through the center of the defect in the coronal plane of the sample. The sections were stained with HE staining and analyzed with an optical microscope (Olympus, Japan). The area of new bone formation (NBF) was quantified in percentage using the Image J software (Version 1.8.0, USA). Tartrate-resistant acid phosphatase (TRAP) and alkaline phosphatase (ALP) staining of the paraffin sections was also performed using the TRAP/ALP double stain kit (Wako, Japan) according to the manufacturer’s instructions. The number of TRAP positive cells was counted independently by two authors (H.P. and Z.W.) in a blinded manner, and the mean value was applied for analysis. For quantification of ALP staining sections, the mean density (MD) was measured by the Image-Pro Plus software (Version 6.0, USA). The method of measurement was described briefly in the following: (a) images were saved in TIFF format and then performed optical density correction to remove background interference; (b) selected the areas of interest (AOI) using the irregular AOI tools; (c) selected colors for count by the histogram-based method; (d) get the stained area (SA) value and integrated optical density (IOD) value and then calculate the mean density (MD) value (MD=IOD/SA).

### Immunohistochemical evaluation

For immunohistochemistry, the activity of endogenous peroxidase was quenched with 3% hydrogen peroxide at room temperature for 15 min. Antigen retrieval was then performed with citrate buffer at 80 °C for 10 min for OCN and VEGF immunohistochemistry detection. Primary antibody against OCN (1:200, 23418-AP; Proteintech, China) and VEGF (1:200, 19003-AP; Proteintech, China) was used for 1 h. As a secondary antibody, anti-rabbit IgG linked to horseradish peroxidase was used for 1 h, then stained by streptavidin-peroxidase (SP) for 30min. Afterward, slices were washed with PBS for 3 times, and color was rendered by DAB for 10 min. The sections were examined under light microscopy (Olympus, Japan). The MD values were calculated by the Image-Pro Plus software (Version 6.0); the measure method was consistent with the above.

### Statistical analysis

Descriptive statistics were presented as the mean ± standard deviation (SD). Specific differences between groups were assessed using independent sample pairwise t-tests. For multi-group comparisons, one-way analysis of variance (ANOVA) was conducted and followed by LSD-t multiple comparison test. All statistical analyses were performed using the SPSS 17.0 statistical software (SPSS Inc., USA), and *p < 0.05* was considered statistically significant.

## Results

**Fig. 1:**
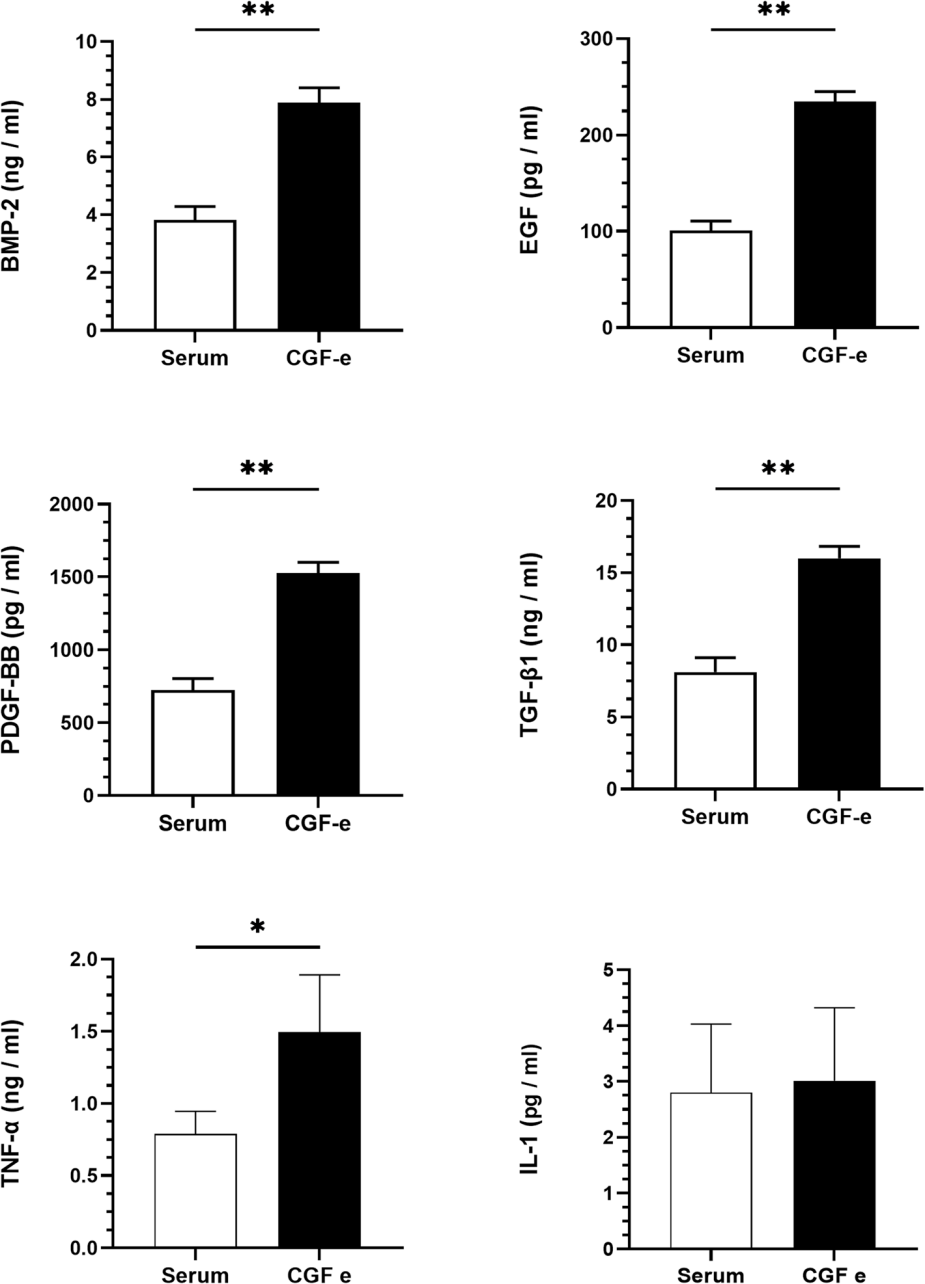
Determination of growth and inflammatory factors in CGF-e and serum. *p < 0.05; **p < 0.01

The concentrations of growth factors and inflammatory factors in serum and CGF-e are shown in Fig. 1. The contents of BMP-2, EGF, PDGF-BB, and TGF-β1 in CGF-e were significantly higher than those in serum (*p < 0.01*). The contents of TNF-α in CGF-e were significantly higher than those in serum (*p* < 0.05); the contents of IL-1 in CGF-e and serum had no significant difference (*p > 0.05*).

**Fig. 2:**
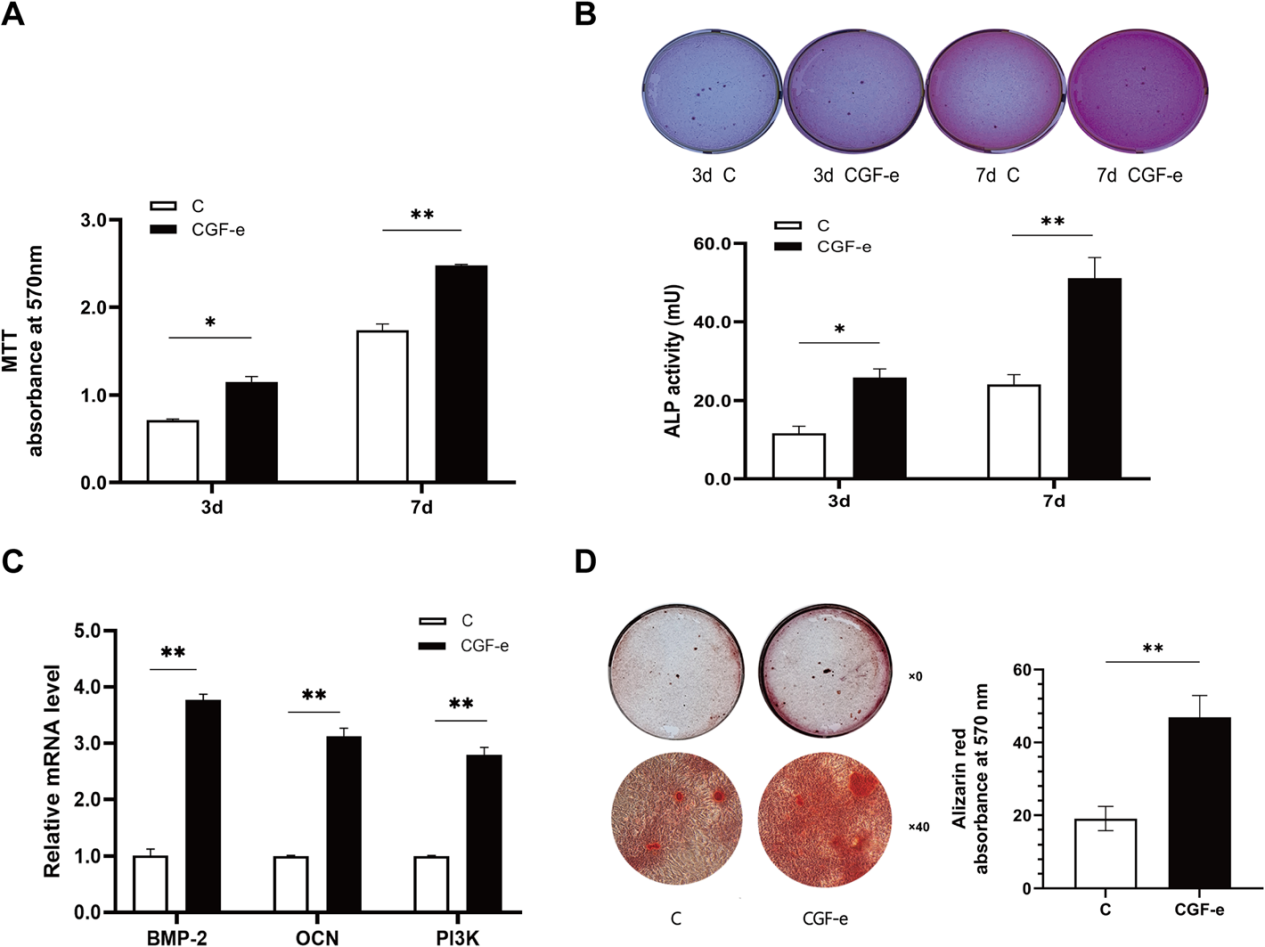
Effects of CGF-e on osteoblast proliferation and differentiation. **a** Proliferation analysis of MC3T3-E1 cells, the optical density value of CGF-e group was significantly higher than control group at 3 days. **b** ALP staining and enzyme activity of MC3T3-E1 cells, the CGF-e group exhibited more ALP-stained spot than control. Pictures of the ALP staining plates were taken using a Canon 60d camera (no magnification). Similarly, the enzyme activity in the CGF-e group was significantly higher than control at 3 days. **c** Relative gene expression of MC3T3-E1 cells, the relative gene expression of the two genes was significantly higher in the CGF-e group compared with the control group (BMP-2, bone morphogenetic protein-2; OCN, osteocalcin; PI3K, phosphoinositide 3-kinase). **d** Mineralization analysis of MC3T3-E1 cells, the number of calcium nodules of the CGF-e group was more than the control group. Pictures of the Alizarin Red staining plates were taken using a Canon 60d camera (no magnification); the samples were then examined under a light microscope at ×40 magnification. Quantitative analyses showed that absorbance value of CGF-e group was higher than control. *p < 0.05; **p < 0.01

CGF-e enhanced the proliferation and osteogenic differentiation of MC3T3-E1 cells. The MTT assay results indicated that the optical density value of CGF-e group was significantly higher than the control group at 3 days (*p < 0.05*) and 7 days (*p < 0.01*) (Fig. 2a). The ALP staining showed that the CGF-e group exhibited more ALP-stained spot than control. Similarly, the ALP activity in the CGF-e group was significantly higher than control at 3 days (*p < 0.05*) and 7 days (*p < 0.01*) (Fig. 2b). As shown in Fig. 2c, the relative gene expression of BMP-2 and OCN in CGF-e group was obviously higher than the control (*p < 0.01*). Alizarin Red staining showed that the number of calcium nodules of the CGF-e group was more than the control group. Quantitative analyses indicated that the absorbance value of the CGF-e group was higher than the control group (*p < 0.01*) (Fig. 2d).

**Fig. 3:**
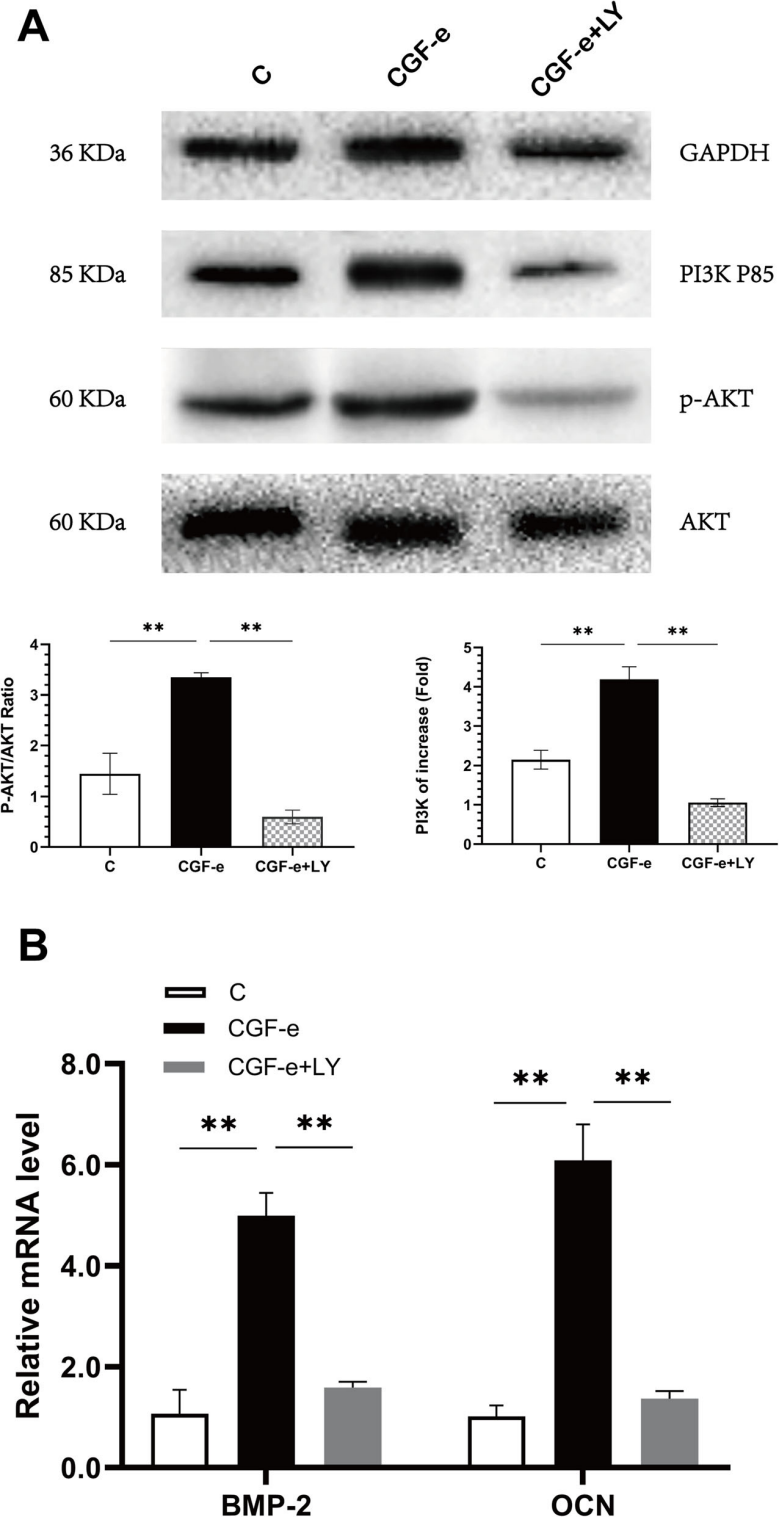
Role of PI3K/AKT signaling pathway. **a** Relative protein expression of MC3T3-E1 cells. Results showed that the protein expression of CGF-e group was significantly higher than the control group. **b** Relative gene expression level of BMP-2 and OCN after the PI3K/AKT was blocked with the PI3K inhibitor LY294002. Results showed that the gene expression of CGF-e+LY294002 group was significantly lower than the CGF-e group. *p < 0.05; **p < 0.01

CGF-e promoted osteogenic differentiation of MC3T3-E1 cells through the PI3K/AKT signaling pathway. As shown in Fig. 2 c and Fig. 3 a, the relative gene and protein expressions of PI3K and p-AKT of the CGF-e group were obviously higher than the control group (*p* < 0.01). Notably, the facilitation effect of CGF-e was suppressed by the PI3K inhibitor LY294002; the relative gene expression level of PI3K, AKT, BMP-2, and OCN of the CGF-e+LY294002 group was significantly lower than the CGF-e group (*p < 0.01*) (Fig. 3).

**Fig. 4:**
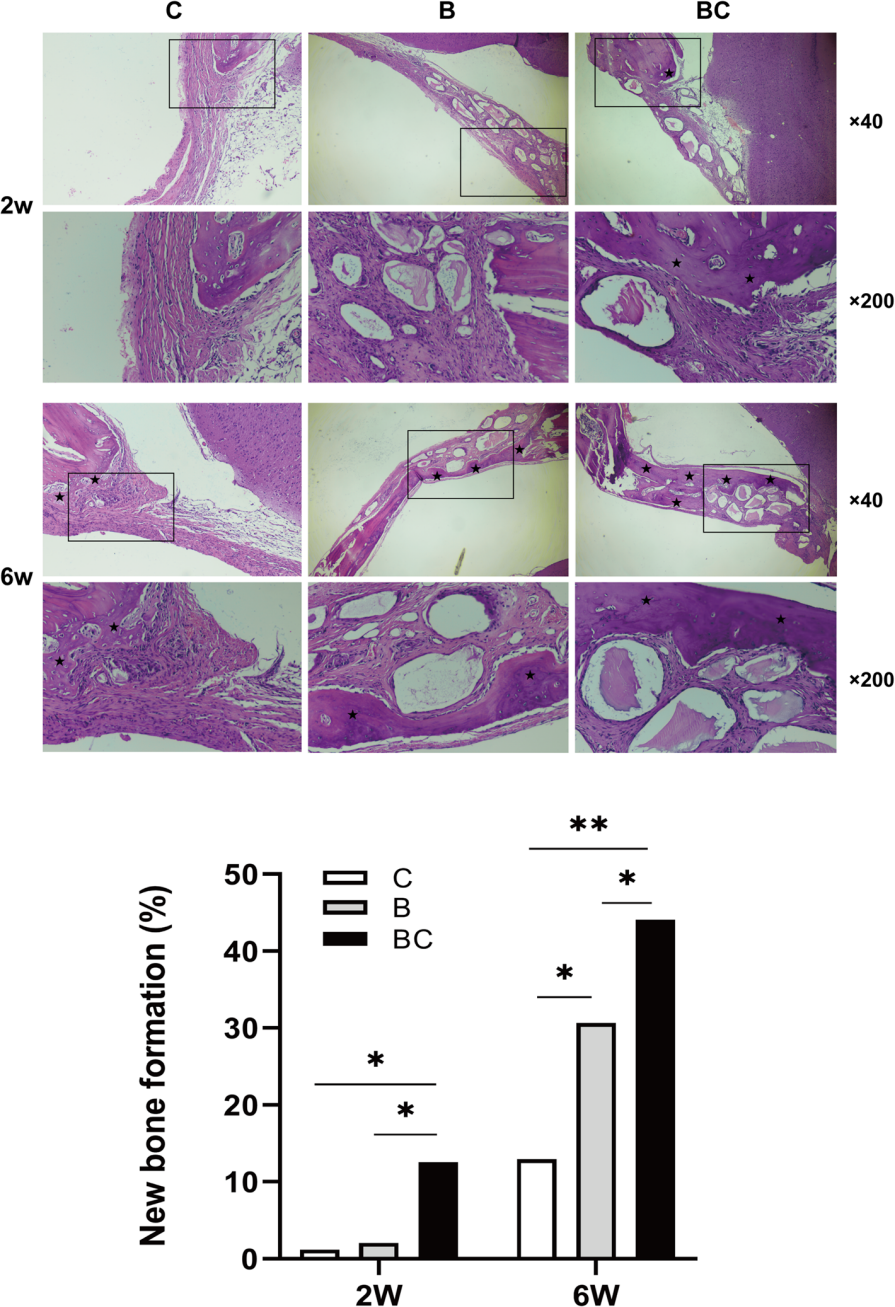
HE staining analysis of the bone regeneration of critical-sized rat cranial defects. At 2 weeks, loose fibro-collagenous stroma and irregularly arranged cells could be seen at the edge of defect area in groups B and C, while new bone formation could be observed in group BC, which was limited to the area of the defect margin. At 6 weeks, only a small amount of new bone could be seen in group C; in group B, obvious new bone formation was seen, extending along the edge of the bone graft material; in group BC, a large amount of new bone formation was seen, extending from the old bone defect to the center of the bone defect, surrounding bone graft material. Group C, unfilled defect for control; group B, bone collagen alone; group BC, CGF-e + bone collagen. The histological images were photographed under a light microscope at ×40 and ×200 magnifications. (Black asterisk: new bone tissue) Quantitative analysis showed that the area of new bone formation (NBF) of the BC group was significantly higher than C and B groups. *p < 0.05; **p < 0.01

**Fig. 5:**
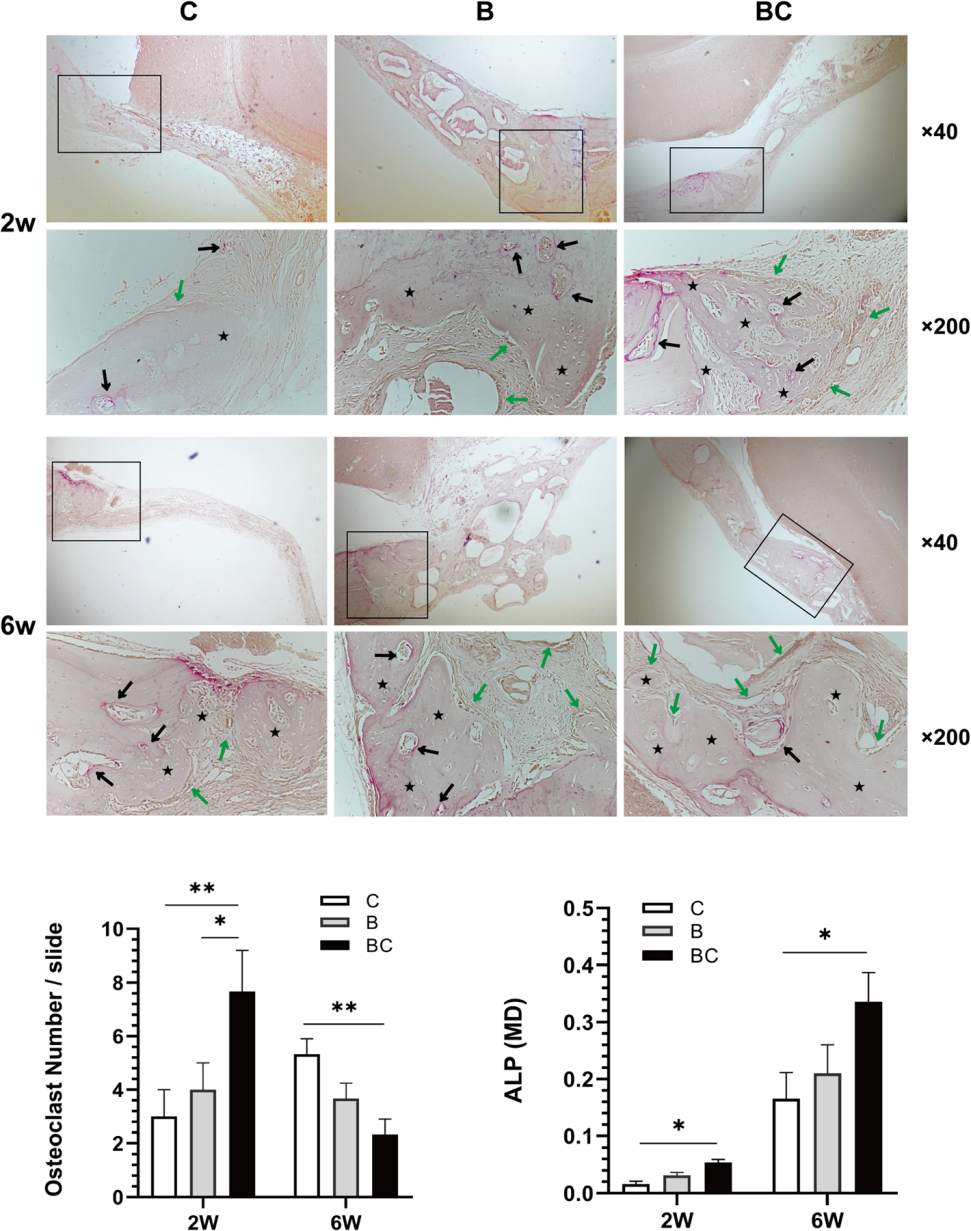
TRAP/ALP staining analysis. The images were photographed under a light microscope at ×40 and ×200 magnifications. (Black asterisk, new bone tissue; black arrows, TRAP positive osteoclasts which were stained purple; green arrows, ALP-positive osteoblasts which were stained brown) For quantification of TRAP staining, the mean of number of positive cells was determined from three serial sections; for quantification of ALP staining, the mean density (MD) was measured. At 2 weeks, the osteoclast number and the MD of ALP in group BC were significantly higher than that of groups C and B; at 6 weeks, the osteoclast number of group BC was obviously lower than that of groups C and B; conversely, the mean density (MD) of ALP in group BC was significantly higher than C and B groups. *p < 0.05; **p < 0.01

**Fig. 6:**
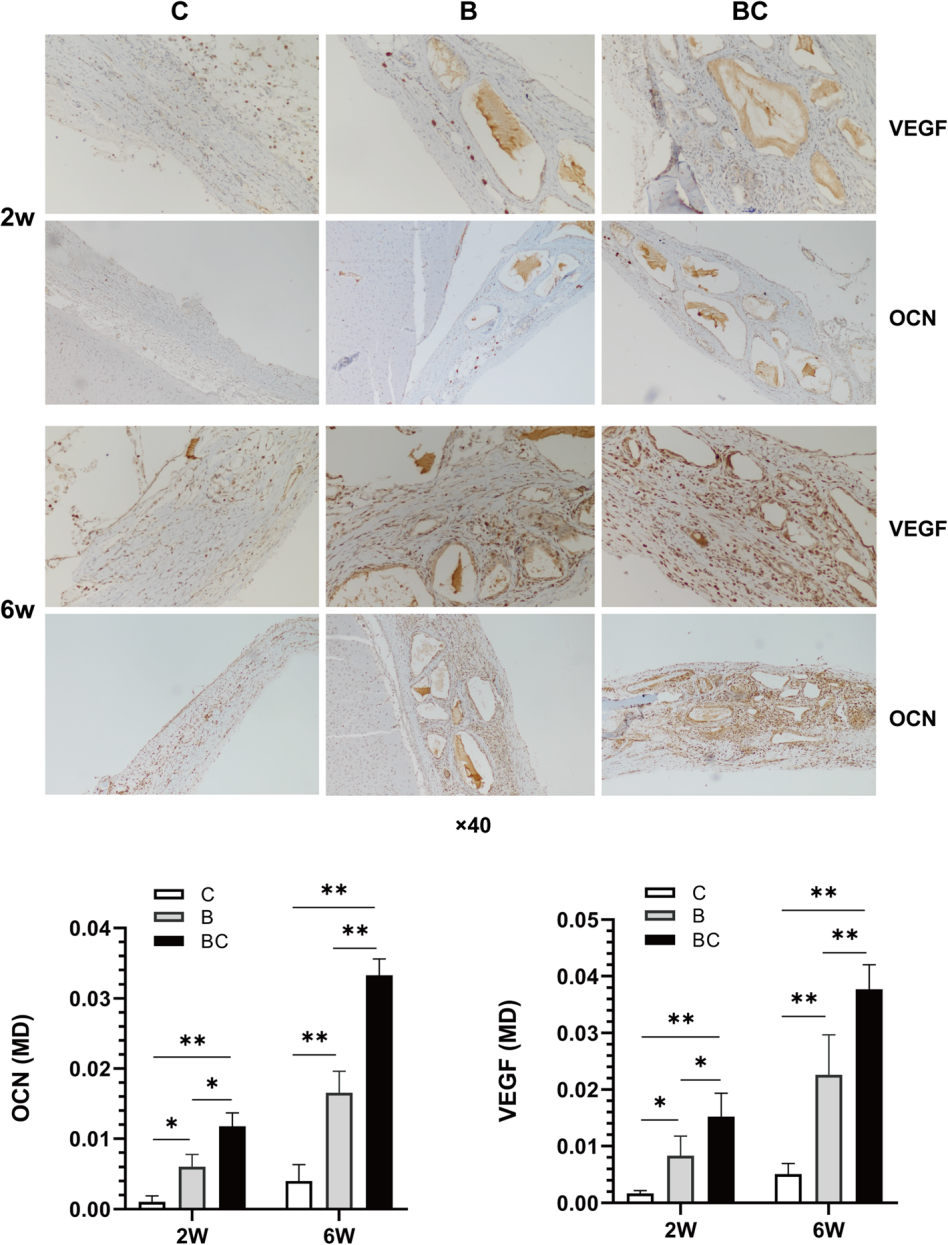
Immunohistochemical analysis of the expression of VEGF and OCN. At 2 weeks, immunohistochemistry revealed weak intercellular expressions of VEGF and OCN in groups C and B; in group BC, the expressions were more obvious. At 6 weeks, obvious intercellular expressions of VEGF and OCN could be seen in groups C and B; in group BC, the cells were strongly positive for VEGF and OCN. The immunostaining sections were observed under a light microscope at ×200 magnification. Group C, unfilled defect for control; group B, bone collagen alone; group BC, CGF-e + bone collagen. Quantitative analysis showed that the mean density (MD) of VEGF and OCN of BC group was significantly higher than C and B groups. *p < 0.05; **p < 0.01

CGF-e enhances the bone regeneration of critical-sized rat cranial defects. As shown in Fig. 4, at 2 weeks, loose fibro-collagenous stroma and irregularly arranged cells could be seen at the edge of defect area in groups B and C, while new bone formation could be observed in group BC, which was limited to the area of the defect margin. At 6 weeks, only a small amount of new bone could be seen in group C; in group B, obvious new bone formation can be seen, extending along the edge of the bone graft material; in group BC, a large amount of new bone formation can be seen, extending from the margin of old bone to the center of the bone defect, enclosing the bone graft material. As shown in Fig. 5, at 2 weeks, the osteoclast number and the MD value of ALP in group BC were significantly higher than that of groups C and B; at 6 weeks, the osteoclast number of group BC was obviously lower than that of groups C and B; conversely, the MD value of ALP in group BC was significantly higher than C and B groups. As shown in Fig. 6, at 2 weeks, immunohistochemistry revealed weak intercellular expressions of VEGF and OCN in groups C and B; in group BC, the expressions were more obvious. At 6 weeks, obvious intercellular expressions of VEGF and OCN could be seen in groups C and B; in group BC, the cells were strongly positive for VEGF and OCN. Quantitative analysis showed that the expression of VEGF and OCN of BC group was significantly higher than C group (*p < 0.01*) and B group (*p < 0.05*).

## Discussion

Osteoblast attachment and proliferation on dental implant surface is the beginning of osseointegration; various growth factors released from the activated platelets play an important role during the above process [16]. Due to the particular centrifugation procedure, CGF was rich in abundant dense fibers and a significant number of growth factors which were essential for wound healing and bone regenerative [17, 18]. However, in the 5th EAO consensus conference, it was stated that the exact role of platelet concentrates for bone regeneration is not known yet, and there is currently no definitive evidence that platelet concentrates have any additional benefits to traditional surgery [7]. One major reason could be because the in vivo degradation rate of CGF gel is too fast to maintain the effective concentrations of growth factors, namely, the CGF-e plays a major role for localized delivery of growth factors [10, 19]. As described in the previous article [20], all of the growth factors were predominantly stored in the extract of CGF and cooperated with each other to stimulate cell proliferation, matrix formation, and angiogenesis. Studies have also shown that CGF-e with appropriate concentration could promote proliferation and osteogenic differentiation of bone marrow mesenchymal stem cells; however, its excessive concentration could have inhibitory effects [3, 14]. The negative effect might be probably due to the inflammatory cytokines contained in CGF-e, such as interleukin-1b and tumor necrosis factor, which may inhibit osteogenic differentiation and bone formation [21]. According to the previous study [14, 22], 0.5% CGF-e was chosen for this experiment.

As we know, three main types of rejection can occur after xenotransplantation: hyperacute xenograft rejection, acute humoral xenograft rejection, and acute cellular rejection [23]. Among them, immune cells such as T and B lymphocytes, neutrophils, natural killer cells, or myeloid-derived suppressor cells play a pivotal role [24]. In this study, the CGF-e contained mainly growth factors and inflammatory cytokines whereas did not contain any cell cellular component. Currently, little information is currently available on the xenograft rejection phenomenon of CGF-e. Therefore, to evaluate whether CGF-e would induce a xeno-antigen immune rejection, we choose the MC3T3-E1 osteoblast-like cell line. Results showed that the CGF-e prepared from rat blood did not appear to pose any safety concerns for MC3T3-E1 cells. However, the results of in vitro experiments did not adequately represent the complex environment in vivo, as such, we chose rats rather than mice for the in vivo studies.

The PI3K/Akt signaling pathway is closely related to the osteogenic differentiation and bone formation [25]. The present study shows that CGF-e activated the PI3K/AKT signaling to enhance osteogenic differentiation and mineralization of MC3T3-E1 cells, thereby contributing to bone formation in rat cranial defects. This effect of CGF-e might possibly be induced by the growth factors contained in CGF-e, such as PDGF, TGF-β, BMP-2, VEGF, and IGF. For example, numerous previous studies showed that the PI3K/AKT pathway can be activated by PDGF and IGF [26, 27]; TGF-β also interacts with the PI3K/AKT pathway during the wound healing process [28] and promotes the osteoinduction [29]; BMP-2 promotes the differentiation of osteoblasts by activating the PI3K/Akt pathway [30]; VEGF can regulate angiogenesis through the PI3K/AKT pathway [31]; in addition, IRS-1/PI3K pathway regulates IGF-I-induced cell proliferation through AKT signaling [32].

To explore the mechanism underlying the regulation response of CGF-e, we utilized the PI3K inhibitor LY294002 to investigate the changes of osteogenic differentiation mediated by CGF-e. This study showed that the high protein expression levels of PI3K and p-AKT as well as the high gene expression levels of BMP-2 and OCN were significantly suppressed by LY294002, indicating that the CGF-induced osteogenic differentiation was associated with the PI3K/AKT pathway. This result was consistent with the previous literature. McGonnell et al. [33] demonstrated that PI3K/Akt signaling pathway is essential for osteogenesis. Peng et al. [34] demonstrated that Akt-knockdown resulted in the inhibition of endochondral ossification. Similarly, Ulici et al. [35, 36] reported that longitudinal bone growth is suppressed by PI3K inhibitor LY294002.

In agreement with the in vitro findings, the combination of CGF-e and bone collagen promoted bone regeneration in the critical-sized rat cranial defects. However, as shown in Fig. 4, the Bio-Oss granules in groups B and BC are all surrounded by the fibrous tissue; the reasons may be as follows: (1) fibrous connective tissue invasion due to periosteum tears during suture; (2) inflammatory reaction due to bacterial contamination during surgery; (3) the bone matrix was not formed completely due to the relatively short time of observation. Interestingly, the TRAP/ALP staining showed that the number of osteoclasts in BC group was the highest at 2 weeks, while it is the lowest at 6 weeks. This suggested the positive bone remodeling process in the early stage, attributable to the osteogenic effects of CGF-e. After 6 weeks, the bone remodeling of the BC group was almost completed; this thereby reduced the activity and survival of osteoclasts to reduce osteoclastic bone resorption. During this process, the ALP expression of osteoblasts in the BC group was consistently highest; this indicated that the CGF-e has been at play in the bone regeneration process.

## Conclusions

In summary, we demonstrated the following: (1) CGF-e activated the PI3K/AKT signaling pathway to enhance osteogenic differentiation and mineralization of MC3T3-E1 cells in vitro; (2) CGF-e promoted the bone formation of rat cranial defect model in vivo. However, the limitations of the present study are as follows: (1) without a detailed compositional analysis of CGF-e, particularly the constituents of immune complexes, such as antigens and other ingredients; (2) further work is required to determine the specific mechanisms involved; in addition, further exploration and optimization of CGF-e are required.

## Data Availability

The datasets used and/or analyzed during the current study are available from the corresponding author on reasonable request.

## Abbreviations

CGF: Concentrated growth factors
CGF-e: Concentrated growth factor extract
GAPDH: Glyceraldehyde-3-phosphate dehydrogenase
BMP-2: Bone morphogenetic protein-2
OCN: Osteocalcin
PI3K: Phosphoinositide 3-kinase
AKT: Serine threonine kinase
p-AKT: Phosphorylation of AKT
